# *Ab initio* electronic properties of dual phosphorus monolayers in silicon

**DOI:** 10.1186/1556-276X-9-443

**Published:** 2014-08-28

**Authors:** Daniel W Drumm, Manolo C Per, Akin Budi, Lloyd CL Hollenberg, Salvy P Russo

**Affiliations:** 1 Theoretical Chemical and Quantum Physics, School of Applied Sciences, RMIT University, Melbourne, VIC 3001, Australia; 2 School of Physics, The University of Melbourne, Parkville, VIC 3010, Australia; 3 CSIRO Virtual Nanoscience Laboratory, 343 Royal Parade, Parkville, VIC 3052, Australia; 4 Now at NanoGeoScience, Nano-Science Centre, University of Copenhagen, Universitetsparken 5, København Ø 2100, Denmark

**Keywords:** *Ab initio*, Density functional theory, Bilayers, Phosphorus in silicon

## Abstract

In the midst of the epitaxial circuitry revolution in silicon technology, we look ahead to the next paradigm shift: effective use of the third dimension - in particular, its combination with epitaxial technology. We perform *ab initio* calculations of atomically thin epitaxial bilayers in silicon, investigating the fundamental electronic properties of monolayer pairs. Quantitative band splittings and the electronic density are presented, along with effects of the layers’ relative alignment and comments on disordered systems, and for the first time, the effective electronic widths of such device components are calculated.

## Background

We are currently living through a transition in electronic circuitry from the classical to the quantum domain. With Moore’s Law on the way out, thanks to the recent unveiling of ohmic 2 nm epitaxial nanowires [1] and epitaxially gated single-atom quantum transistors [2], the challenge for scientists becomes finding new ways to increase the density and speed of devices as we can no longer rely on being able to shrink their components.

Far-sighted speculation has already been abundant for many years regarding efficient use of the third dimension in device architecture [3-6], breaking the two-dimensional paradigm of current electronics manufacturing techniques. Recent germanium-based works [7,8] illustrated fundamental physics required for full 3D device implementation and heralded the creation of multiple stacked *δ*-layers of dopants within a semiconductor. Each of these layers could potentially display atomically abrupt doped regions for in-plane device function and control. Multiple layers of this nature have indeed been created in Ge [9]. The P in Ge atomic layer deposition technique parallels phosphorus in silicon 1/4 monolayer (ML) doping (Si: *δ*P), created using scanning tunnelling microscope lithography, with a few minor technological improvements (annealling temperatures, amongst others) [8]. In contrast, one major advantage of improvements to silicon technology is that uptake may be far easier, given the ubiquity of silicon architecture in the present everyday life. We may therefore expect to see, in the near future, Si: *δ*P systems of similar construction.

The time is thus ripe to attend to possible three-dimensional architectures built from phosphorus in silicon. Although Si:P single-donor physics is well understood, and several studies have been completed on single-structure epitaxial Si: *δ*P circuit components (such as infinite single monolayers [10-17], single thicker layers [18,19], epitaxial dots [20], and nanowires [1,21]), a true extension studying interactions between device building blocks in the third dimension is currently missing.

The description of experimental devices is a thorny problem involving the trade-off between describing quantum systems with enough rigour and yet taking sufficient account of the disorder inherent to manufacturing processes. A first approach might therefore be to study the simplest case of interacting device components, namely two P-doped single monolayers (bilayers) [22,23]. Given the computational limitations of *ab initio* modelling it is currently not possible to treat the disordered multi-layer system in full. Two approaches suggest themselves. In [23] the approach was to simplify the description of the delta-layer in order to study disorder through a mixed atom pseudopotentials approach. Here, we instead develop a rigorous model of an idealised, perfectly ordered multi-layer system in order to make connections to an understanding gleaned from both the mixed-atom approach and from other idealised models. The two approaches are complementary: alone, neither achieves a complete description, but together, they offer good comparisons from which one may draw the firmest conclusions available regarding experimental devices. The second approach, dwelt upon in this work, also offers descriptions of systems that should become available with improvements to the manufacturing processes mentioned above. As such, this is the focus of our discussion.

**Figure 1:**
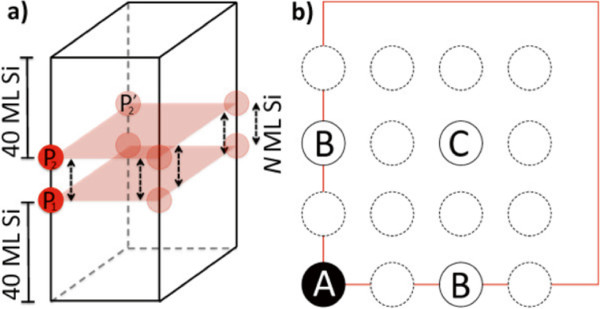
**Model schematics. (a)** Type-*A* bilayer system: tetragonal cell (lines), donors (P _1_, P _2_), periodic images (translucent circles), and effective donor layers (translucent sheets). Varying separation within bilayers (arrows). **(b)** Second-layer dopant (in-plane) positions: P _1_ projection (black circle), coplanar Si atoms (circles), type-*A*, -*B*, and -*C* positions, other monolayers’ atoms’ projections (dashed circles), and periodic boundary (square).

Whilst single-monolayer studies converge properties by increasingly isolating the layers [11,14,16], at closer separations, it is impossible to divorce specific interactions between two layers from those between all of their (infinite) periodic replications. Further, effects arising due to atomic-scale mismatches in each layer’s doping locations cannot be seen when the neighbouring layer is a perfect replica. Building upon the methodology established whilst investigating single *δ* layers [16], expanded upon when considering thicker layers comprised of multiple adjacent *δ* layers [19], and further extended to consider *δ*-doped nanowires [21], here, we model Si: *δ*P bilayers, varying both their vertical separation (Figure 1a) and their relative in-plane alignment (Figure 1b).

## Methods

*δ* layers of P are created on Si (001) terraces before being epitaxially coated with further Si [24-27]. It is easy to envision this coating process being monitored and halted at a desired buffer thickness, before a new *δ* layer of P is created (and/or patterned). Single *δ* layer findings [16] suggest that layers interact when less than 80 monolayers (approximately 10.9 nm) of silicon separate them, and that at 80 ML, their properties converge with respect to silicon cladding depth. In that model, periodic replications of the layers were identical by construction, with no possibility of any deviation. Here, we explicitly allow for such differences by including a second layer in the model.

*c*(2×2) cells including two *δ*-layers at *N* ML separation and 80 ML of Si cladding were built (*N* ∈ {4,8,16,40,60,80}). Doping into a new layer can be accomplished at several locations [19]. For *N*mod(4) = 0 systems, this can occur in three ways (Figure 1b): directly above the original dopant (type *A*), at either position nearest *A* in the plane (type *B*), or at maximal in-plane separation (type *C*). Note that *B* is not the nearest neighbour of either *A* or *C* in the silicon lattice (see Figure 1b).

We note that *N*mod(4) ∈ {1,2,3} systems exhibit new position types, requiring further modelling. Although such investigation would greatly inform the ongoing discussion of disorder in *δ*-doped systems, due to computational resource constraints, they are not considered here.

Models were replicated as *A*_*N*_, *B*_*N*_, *C*_*N*_, and undoped (for bulk properties comparison without band-folding complication) structures. Electronic relaxation was undertaken, with opposite donor spins initialised for each layer and various properties calculated. The general method of [16] using SIESTA[28], and energy convergence of 10^-6^ eV, was used with two exceptions: an optimised double- *ζ* with polarisation (DZP) basis [19] (rather than the default) was employed for all calculations, and the *C*_80_ model was only converged to 2 × 10^-4^ in density (and 10^-6^ eV in energy) due to intractability. Band structures had at least 25 points between high-symmetry locations.

The choice of a DZP basis over a single- *ζ* with polarisation (SZP) basis was discussed in [16], where it was found for single *δ* layers to give valley splittings in far better agreement with those calculated via plane-wave methods. In the recent study by Carter et al. [23], less resource-intensive methods were employed to approximate the disordered-bilayer system, however, here we employ the DZP basis to model the completely ordered system.

## Results and discussion

### Benchmarking of *N* = 80 model

Although we used the general method of [16], as we used the optimised basis of [19], we benchmark our *A*_80_ model with their 80 ML single- *δ*-layer (*δ*_1_) calculation rather than those of [16]. (Lee et al. [18] also used the same general method.) Our supercell being precisely twice theirs, apart from having spin freedom between layers, results should be near identical.

**Figure 2:**
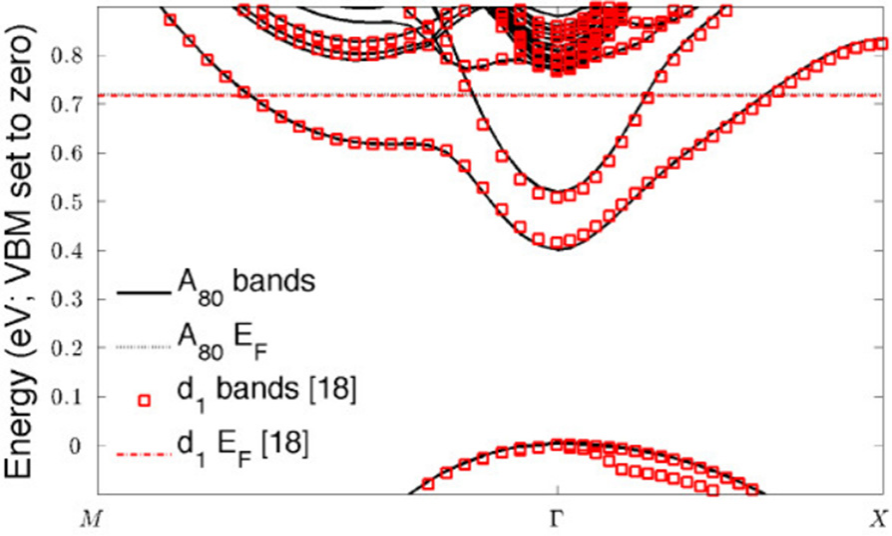
***A***_**80**_** band structure and the** ***δ***_**1**_** band structure of **[12]**.** The partially occupied bilayer bands are doubly degenerate, and the valence band maximum has been set to zero energy.

Figure 2 is the *A*_80_ band structure. Agreement is very good; band shapes are similar, and the structure is nearly identical. A closer look reveals that *A*_80_ has two bands to the *δ*_1_’s one, as we should expect – *A*_80_ has two dopant layers to *δ*_1_’s one. Due to 80 ML of Si insulation, the layers behave independently, resulting in degenerate eigenspectra. Comparison of band minima shows quantitative agreement within 20 meV; the discrepancy is likely a combination of numerical differences in the calculations (generally accurate to approximately 5 meV), the additional spin degree of freedom (which may allow less repulsion between the layers), and band folding from the extension of the bilayer supercell in *z*.

### Band structures and splittings

Band structures for other models were calculated in the same fashion. Comparisons of band minima are shown in Table 1. Within types, the band minima change drastically as *N* shrinks and the *δ* sheets come closer together. The natural progression of this is to the *δ*_2_ results [19], where two layers are directly adjacent (although the location of the dopant in the second layer will be different, as mentioned above, due to the nature of the silicon lattice).

**Table 1:** Bilayer models’ band minima energies, Fermi levels, and differences between band minima

**Model (type**_***N***_**)**	**Band minima (at Γ, meV)**				**Differences (meV)**				**E**_**F**_** (meV)**
					**Type 1**		**Type 2**		
	**1**	**2**	**3**	**4**	**2 -1**	**4 -3**	**3 -1**	**4 -2**	
*A*_80_	397	397	515	515	0	0	119	119	720
*A*_60_	397	397	516	516	0	0	119	119	720
*A*_40_	397	397	516	516	0	0	119	119	721
*A*_16_	403	421	524	533	18	9	121	112	758
*A*_8_	377	417	498	605	40	107	122	188	761
*A*_4_	323	371	615	652	48	37	291	281	771
*B*_80_	396	396	515	515	0	0	119	119	720
*B*_60_	397	397	516	516	0	0	119	119	720
*B*_40_	397	397	516	516	0	0	119	119	721
*B*_16_	410	410	522	532	0	10	112	122	758
*B*_8_	374	460	505	604	86	99	131	144	765
*B*_4_	340	357	602	649	17	47	262	292	772
*C*_80_	396	397	515	515	0	0	119	119	720
*C*_60_	397	397	516	516	0	0	119	119	720
*C*_40_	397	397	516	516	0	0	119	119	721
*C*_16_	411	414	524	535	3	11	113	121	758
*C*_8_	375	438	488	591	62	103	112	153	758
*C*_4_	180	413	608	710	233^a^	102	428^b^	299	774

Bands are labelled counting upwards from the conduction band minimum, and the valence band maxima have been set to zero energy. ^a^This value is far more in keeping with the *A*_4_ and *B*_4_ band 3 ***-*** 1 differences, suggesting that the bands may have crossed. ^b^This value should be interpreted as belonging to the 2 -1 column in place of the value marked with ^a^.

**Figure 3:**
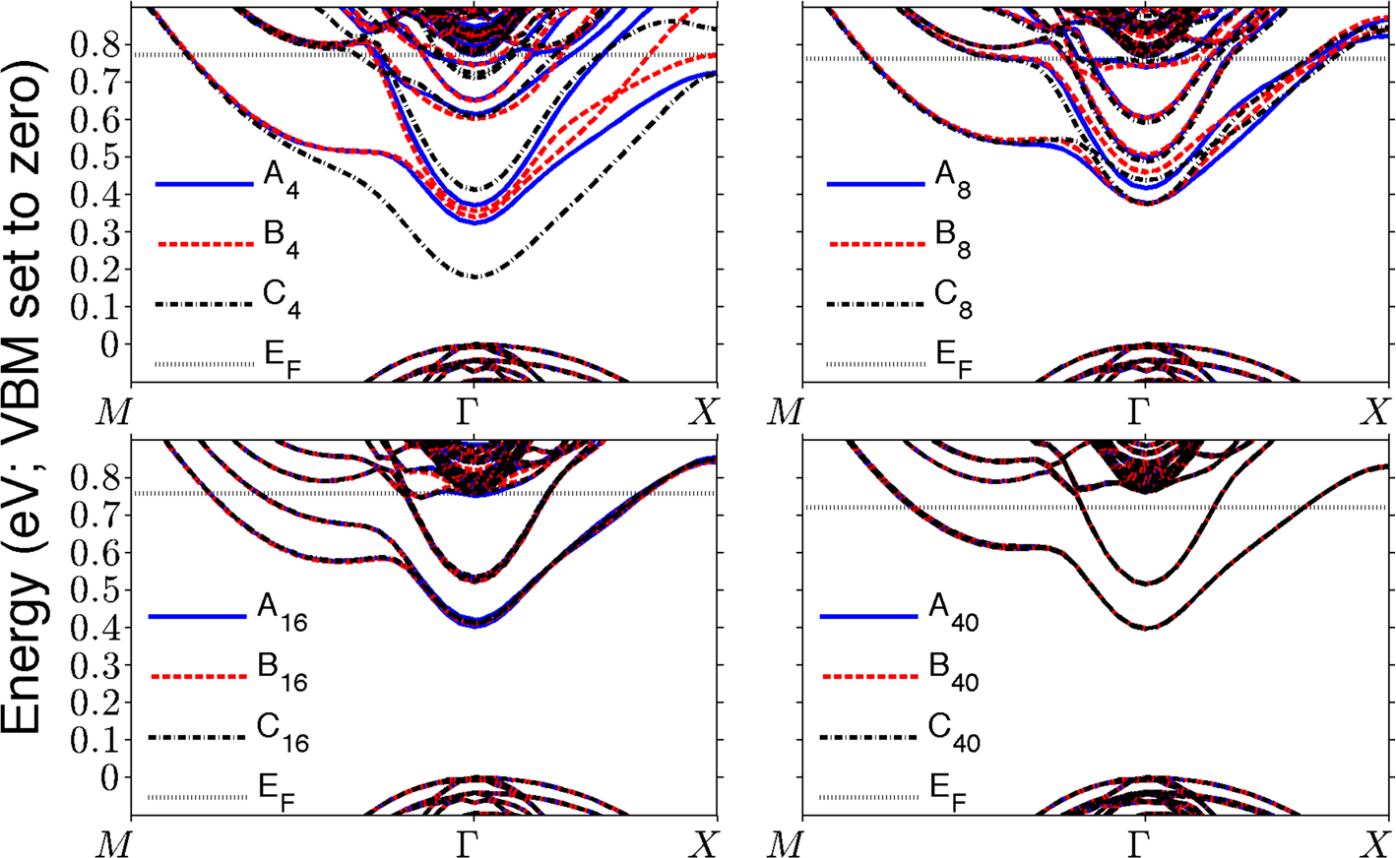
**Band structure of *N *≤ 40 models, from *M *to Γ to *X *.** The valence band maxima have been set to zero energy.

In the large-separation limit (*N* ≥ 40), the values across types (same *N*) are quite similar. The full band structures (60, 80 not shown here) are effectively identical from the valence band maximum (VBM) to well above the Fermi level. We focus upon the occupied spectra from VBM to *E*_*F*_: as *N* decreases, differences due to small changes in donor position become apparent. In particular, we find (see Figure 3) that the *C*_4_ model exhibits drastically wider splitting between the first two bands than *A*_4_, which in turn is significantly wider than *B*_4_. *N* ≥ 40 models show occupation of four bands; a fifth (with minimum away from Γ) dips below *E*_*F*_ for *N* = 16 and 8. (For *N* = 4, the minimum shifts to be at Γ.) The tetragonal symmetry means that this fifth band is four-fold degenerate, so these models have four further, for a total of eight, channels open for conduction, until they merge by *N* = 4. These fifth bands, however, do not penetrate very far below the Fermi level and are henceforth ignored.

As has been noted before [14,16], the specific ordering of donors and symmetries inherent in (or broken by) their placement have great effect upon band energies. Whilst for single layers, valley splitting was paramount [15,16]; here, we introduce the additional possibilities of Coulombic interaction with far-away dopants and quantum interactions with near-neighbour dopants.

Upon closer inspection, holding too closely to single-layer valley splitting proves to be a somewhat naïve way of discussing of the band structures of Figure 3. When all models are compared from *N* = 80 down, it is easily seen that bands come in pairs in the bilayer models, and therefore, at *N* = 80, the equivalent of single-layer valley splitting is the gap between bands one and three (type 2 in Table 1). Due to their large spatial separation, electrons inhabiting bands one and two will overlap only to a negligible extent and, hence, share the same energy here. (This type 1 separation corresponds to interlayer effects - see ‘Consideration of disorder’ section for further discussion.)

As *N* →4, however, the layers approach and interact; for the *C*-type model, bands two and three quite clearly cross each other, and it is possible that some mixing of states occurs - which might well be utilised for information transfer between circuit components in a three-dimensional device design; consider two wires crossing at close distance (*N* < 16) in order to share a state between them.

In fact, the differences columns of Table 1 show that the valley splitting is not particularly perturbed until the layers are quite close to each other (*A*_4_, *B*_8_, and *C*_4_), whilst bands which are effectively degenerate at *N* = 80 are not for *N* ≤ 16. The layers are interacting, affecting the multi-electronic wavefunction under these close-approach conditions. At *N* = 4, it is currently impossible to say which contributes more to the band structure.

Within the approximate treatment in [23] it was concluded that the valley splitting in the interacting delta-layers is the same as that for the individual delta-layer. Here we find that in the DZP approach the valley splitting of 119 meV for the interacting delta-layers is about 30% larger than for the individual delta-layer [19]. Of course, Carter et al. themselves acknowledge that their reduced basis functions are not complete enough to represent the ideal system; the SZP results on disordered systems could not have predicted such a difference. We therefore suggest that their estimate of splitting of 63 meV be revised upwards somewhat; the 30% difference seen between ideal single and double layers may be thought of as an upper bound, since the influence of disorder may well counter that of introducing the second layer.

### Density of states and conduction

**Figure 4:**
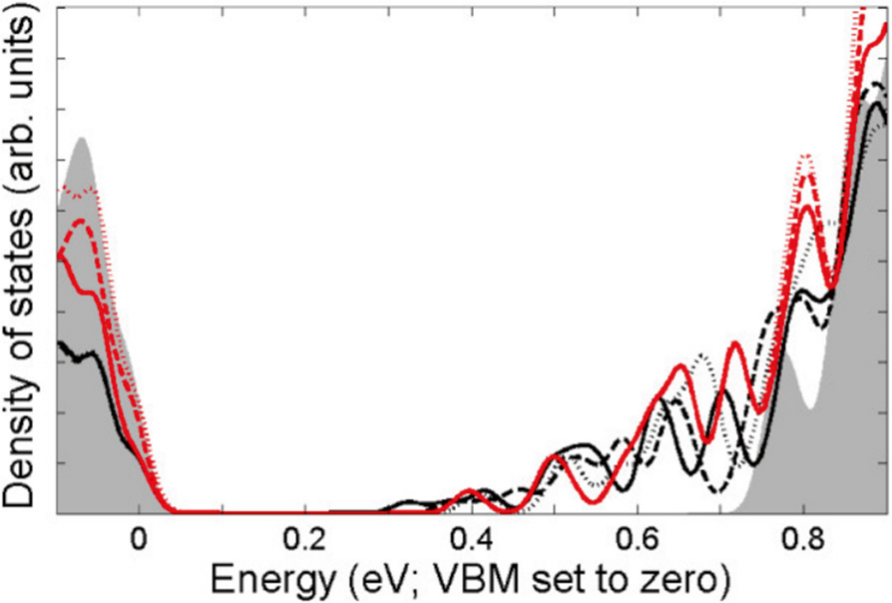
**Densities of states of** ***A***_***N***_** models.** *A*_4_ (solid black), *A*_8_ (dashed black), *A*_16_ (dotted black), *A*_40_ (solid red), *A*_60_ (dashed red), *A*_80_ (dotted red), and bulk (shaded grey). Twenty-five meV Gaussian smearing applied for visualisation purposes.

Figure 4 shows the electronic densities of states (DOS) of the *A*_*N*_ models. As evidenced by the changes in the band minima, lower *N* leads to occupation further into the band gap. In all cases, the occupation is maintained across *E*_*F*_, indicating that the structures are conductive. The DOS of high-*N* models are in good agreement with each other, confirming that these layers are well separated, whilst those of smaller *N* show shifts of density peaks relative to each other and to *A*_80_.

**Figure 5:**
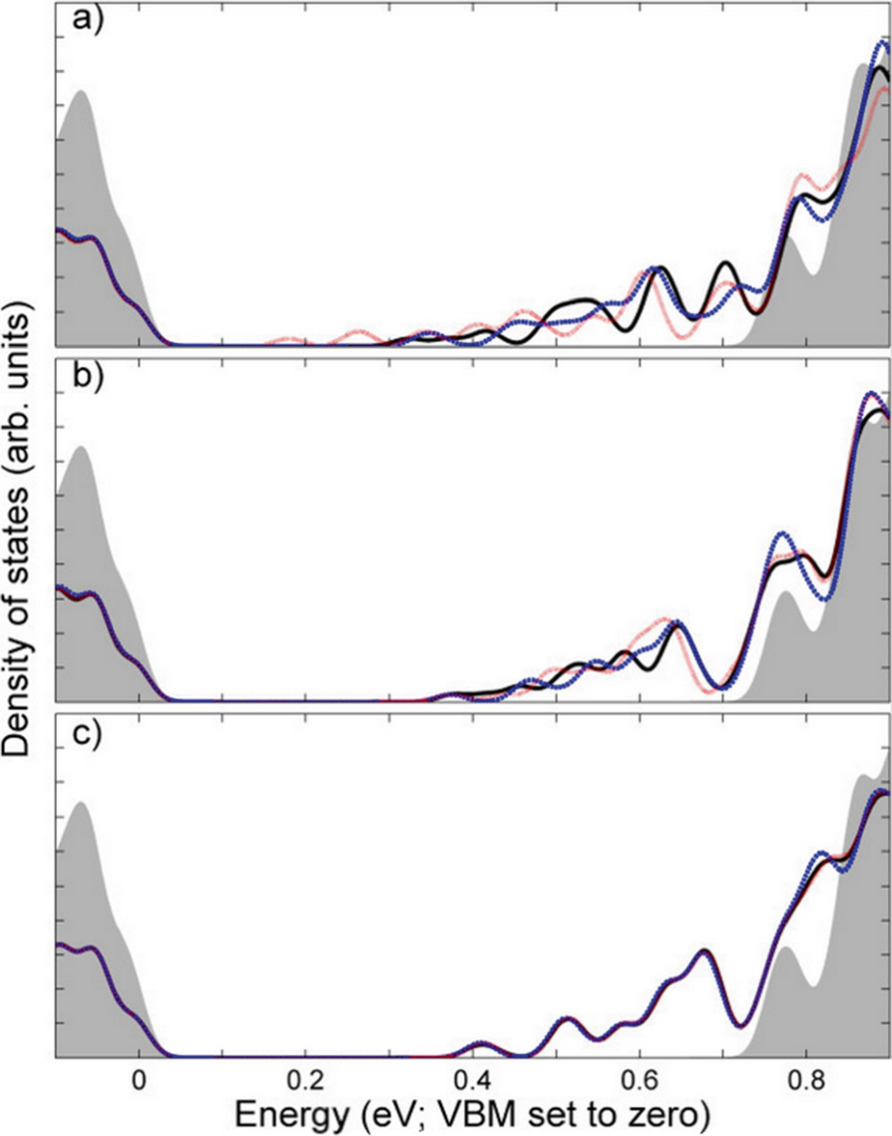
**Densities of states of (a) *N *** **= 4, (b) *N *** **= 8, and (c) *N *** **= 16 models.** Types *A* (black solid lines), type *B* (blue dashed lines), type *C* (red dotted lines), and bulk (grey shaded backgrounds). Energy zero is set to the VBM, Gaussian smearing of 25 meV applied for visualisation purposes.

Less affected by donor placement than the band structure, the DOS shows negligible difference between types by *N* = 16 (Figure 5). Changes between the DOS of *N* = 16-80 models (not shown) therefore arise solely from the inter-layer distance. When one considers the inter-donor separation length, consisting of *N* layers’ separation and a component describing the in-plane separation due to model type, this separation length is far more sensitive to variations of type when the inter-layer separation is short. At *N* = 4, there is already a significant scale difference between the two vector components’ magnitudes which is only exacerbated by increasing *N*.

### The perpendicular electronic cross-section

**Figure 6:**
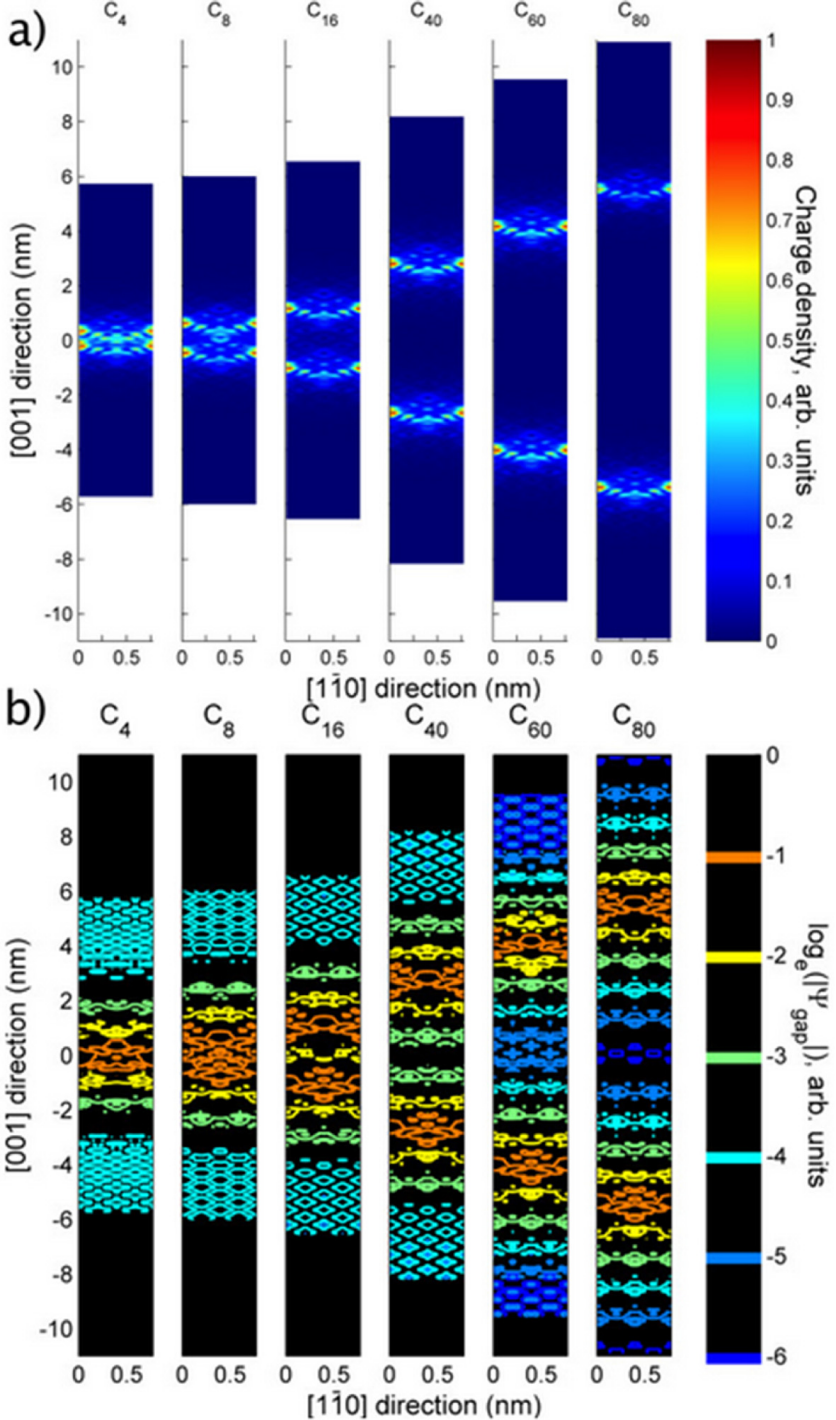
**Local density of states: side view. (a)** Charge density (by LDOS) of *A*_*N*_ models, line-averaged along the [110] direction; **(b)** contour plot of *C*_*N*_ models’ |Ψ_gap_|, maximum along [110] taken for each point. All data normalised to [0,1].

Electronic cross-sections are inferred from the local densities of states (LDOS; integrated from VBM to *E*_*F*_) and may be useful in planning classical devices. *A*_*N*_ models are shown in Figure 6a, where isolation of well-separated and interaction between closely spaced layers are obvious. Significant density overlap begins between *N* = 8 and 16.

Figure 6b depicts the worst-case overlap of the gap-states’ wavefunction (modulus). By *N* = 40, we see (for quantum information purposes) non-negligible overlap (>2%) between the layers. Conversely, *N* ≥ 80 models show that |Ψ_gap_| falls off to less than *e*^-5^. By *N* = 8, |Ψ_gap_| ≥ *e*^-2^ between the layers. This information will be crucial in assessing future quantum device designs.

**Figure 7:**
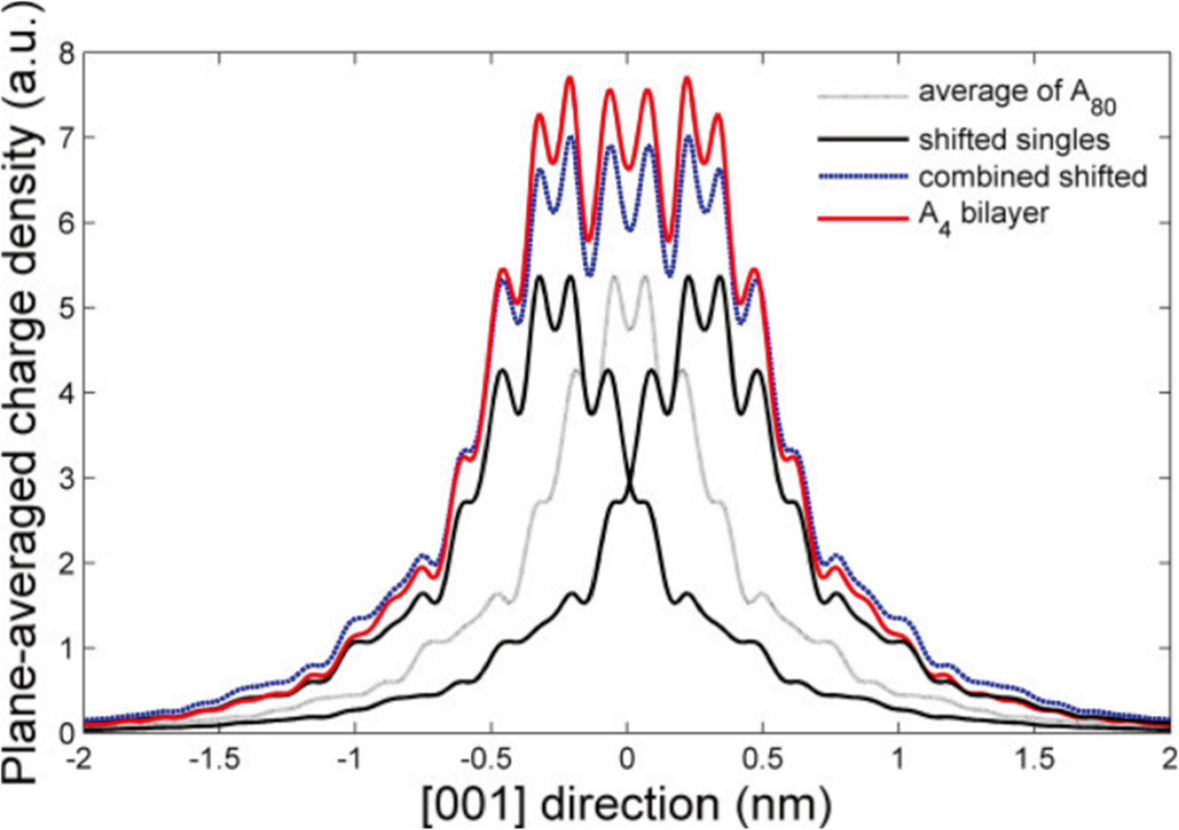
**Single layer versus bilayer density profiles.** Average of *A*_80_ layer profiles about their centers (dotted black), *A*_80_ average profile shifted to center on bilayer positions (solid black), summed shifted profiles (dashed blue), and plane-averaged *A*_4_ profile (solid red). Data were plane-averaged, collapsed to [001], and normalised such that charge density integrated to one.

Interestingly, the falloff from the center of the *N* = 4 model is decidedly similar to the falloff of the well-separated layers of the *N* = 80 model, as Figure 7 illustrates. The bilayer density is slightly higher in the central nanometre and almost negligibly lower in the tail regions. Unlike the *δ*_2_ model [19], which featured doping in two adjacent layers of the Si crystal, the charge density is not pulled inwards much more than a simple combination of two single layers would suggest.

### In-plane density maps

**Figure 8:**
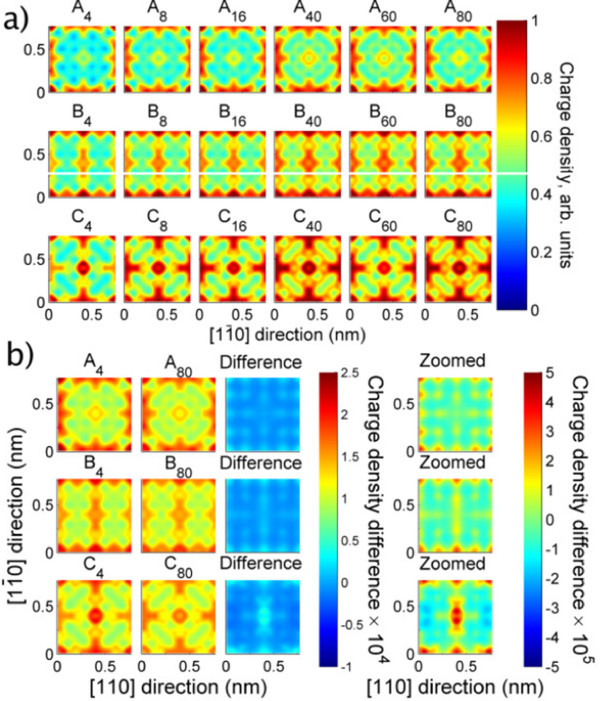
**Local density of states: top-down view. (a)** Charge density (all models), line-averaged along [001] and normalised such that their values’ ranges are each [0,1]. **(b)** Charge densities of *N* ∈ {4,80} models, normalised to |Ψ^2^| = 1. Differences also shown, on two scales.

In-plane density maps will be of interest when considering transport and also when considering disorder. Figure 8a shows the in-plane charge density for all models. In-plane alignment does indeed have a great effect upon the charge density; *A*_*N*_ models exhibit large low-density central regions (away from the donors) whilst *B*_*N*_ have high-density pathways in one direction, and *C*_*N*_ show the greatest extent of high-density regions.

To focus on bilayer-specific effects, *N* = 4 and 80 models were rescaled, and their differences are shown in Figure 8b. The electronic density reorganises as the layers approach, in a type-dependent manner. The magnitude of the rearrangement is ≤ 20% of the single-layer density.

### Consideration of disorder

As mentioned earlier, though the main focus of this work is perfectly ordered systems, recent attention has been given to disorder. Here, we consider how these ordered results can contribute to that discussion. As it is useful to recall which calculations have been previously performed in the literature, Table 2 summarises the state of the field and introduces terminology to distinguish between the various models.

**Table 2:** **Listing of *ab initio * works in this field covering systems with 1/4 ML phosphorus density**

**Model type**		**SZP**	**DZP**
**System**	**Arrangement**		
Bulk		*bulk*-SZP [14,16]	*bulk*-DZP [16]
	Ordered	*δ*-SZP-*ord*[14,16]	*δ*-DZP-*ord*[16,19]
*δ*	Disordered	*δ*-SZP-*dis*[14,23]	*δ*-DZP-*dis*[23]
	Mixed-pseudo	*δ*-SZP-*mix*[14,23]	*δ*-DZP-*mix*[23]
*δ*_*n*∈{2..5}_	Ordered		*δ*_*n*_-DZP-*ord*[19]
	Ordered	*δδ*-SZP-*ord*[23]	*δδ*-DZP-*ord*^a^
*δδ*	Disordered	*δδ*-SZP-*dis*[23]	Intractable
	Mixed-pseudo	*δδ*-SZP-*mix*[23]	
*δ*-wire	Ordered		*δ*-*wire*-DZP-*ord*[21]
	Staggered		*δ*-*wire*-DZP-*stag*[21]

***δ*** refers to a single- ***δ***-layer system, ***δ***_*n*_ to *n* multiple adjacent ***δ*** layers, ***δδ*** to the bilayer systems considered here, and ***δ***-wire to the dually confined monolayer nanowires considered in [21]. Note that further subtleties, such as the vertical separation and in-plane alignment considered here, could form a third (or fourth) tier of model nomenclature, but are omitted for brevity here. ^a^Refers to this work.

Interacting *δ* layers have recently been studied from the point of view that current experimental systems involve some inherent level of disorder [23]. Whilst it is recognised that a complete DZP model of interacting quasi-disordered bilayers is currently intractable (let alone incorporating disorder on any realistic scale), they offered the rational approach of contrasting a DZP model of a single quasi-disordered *δ* layer against an SZP model and then extending the SZP model to cover a quasi-disordered bilayer. The reasonable assumption there was that the differences between SZP and DZP models should be similar in both cases, and the valley splittings of the (missing) DZP model of a quasi-disordered bilayer could thus be inferred. They also considered the approach of using a DZP basis and mixed pseudopotential to describe the disorder; this approach is vastly cheaper computationally and purports to inform us about the splittings due to the presence of the second layer. It is supported by SZP mixed and explicit pseudopotential results in which these interlayer splittings are preserved.

The approach taken in this paper, of calculating the properties of an explicitly ordered bilayer system using a DZP basis, complements that previous work. We can equivalently make comparisons between the ordered single-layer systems of [19] (*δ*-DZP-*ord*) and ordered double-layer systems as calculated with DZP bases here (*δδ*-DZP-*ord*), and between the *δ*-DZP-*ord* systems of [19] and the (DZP) quasi-disordered single-layer system (*δ*-DZP-*dis*) presented in [23], in order to draw inferences about the (intractable, missing) *δδ*-DZP-*dis* model, without at any stage compromising the accuracy of the results by using a less-complete basis set. (We shall now proceed to drop the ‘DZP’ from the labels, since it is ubiquitous here.)

One important point in the consideration of disorder from these ideal models is that, at the lowest separation distances, the crystalline order and alignment of the layers is greatly influencing their band structure. In a disordered system, the alignment effects would largely be negated, or averaged out, since one would expect to encounter all possible arrangements. We therefore limit ourselves to discussing averages of splittings.

The *δ*-*ord* layers show valley splittings (VS) of 92 meV, as compared to the 120(±10%) meV of the *δδ*-*ord* bilayer systems presented here (apart from separations of less than 8 monolayers). The *δ*-*dis* system showed a valley splitting of 63 meV, indicating that we might expect a reduction of valley splitting of up to 32% due to the (partial) inclusion of disorder. We can then infer that the valley splitting in the *δδ*-*dis* systems should be around 81 meV, unless their separations are small (see Table 3).

**Table 3:** Model properties and prediction of disordered splittings

**Separation**	**VS (meV)**	**VS (meV)**	**ILS (Γ, meV)**
**(ML)**	**(*ord*-*δδ*, avg.)**	**(*dis*-*δδ*, est.)**	**(*ord*-*δδ*, avg.)**
80	119	81	0
60	119	81	0
40	119	81	0
16	117	80	9
8	142	97	83
4	309^a^	211^a^	81^a^

The valley splittings are calculated as the average difference between the lower (or upper) of each pair of bands (type 2 from Table 1), whilst the interlayer splittings (ILS) are calculated as the average difference between the lower (or upper) pair of bands (type 1 from Table 1). ^a^These values are likely considerably erroneous due to the crossing of bands in some alignments confusing the averaging of VS and ILS, and the vast effect alignment has at this low separation.

We can estimate the interlayer splitting by taking the differences between bands 1 and 2 and bands 3 and 4 (except at low separation). Averaged values for these are also presented in Table 3. Unfortunately, beyond the SZP models, we have no further information as to the likely behaviour of the *δδ*-*dis* model at the DZP level in this regard, as there can be no interlayer splitting in the isolate single-layer models to compare against.

It is clear from Table 3 that the estimated values for the valley splitting differ from those predicted by the SZP approach (63 meV for all but ‘extremely close separations’). We are in agreement with the finding that narrow separations affect the value greatly. Even allowing for the possibility of overestimation of the valley splitting here (the *δ*-*ord* value was 92 meV) only adjusts the estimated *δδ*-*ord* value by 8 meV, not the 20 required to match the values obtained using the SZP approach.

Obviously, the extension to a full DZP model has brought to light behaviours at small separation not evident from the SZP approach, and further work is required to elucidate these as computational resources improve.

## Conclusions

We have modelled Si: *δ*P bilayers, varying their separation and in-plane alignment. Whilst layers behave independently at large separations (above 40 ML), they interact when brought close together: band structures are affected considerably; variation in the energy splitting between the first two occupied bands for *N* = 4 is considerable, and this variation must be taken into account in any future models of disorder in such closely spaced layers; in-plane charge densities shift by ≤20%. Out-of-plane charge densites overlap to varying extent; wavefunction moduli are more sensitive. For 8 ≤ *N* ≤ 16, four new conduction channels open, making eight in total. Consequences for device design will depend heavily on the desired purpose; detailed information has been presented for several possible issues to facilitate successful design and operation of future three-dimensional devices, be they classical or quantum in nature. Finally, despite single- *ζ* with polarisation results indicating that valley splittings are the same in single- and double- *δ*-layered systems, our results indicate otherwise at double- *ζ* with polarisation level (previously shown to be adequately complete), with implications for the ongoing discussion of disordered systems of this type.

## Competing interests

The authors declare that they have no competing interests.

## Authors’ contributions

DWD, MCP, and LCLH planned the study. DWD, MCP and AB performed the calculations. All authors analysed the results and wrote the manuscript. All authors read and approved the final manuscript.
